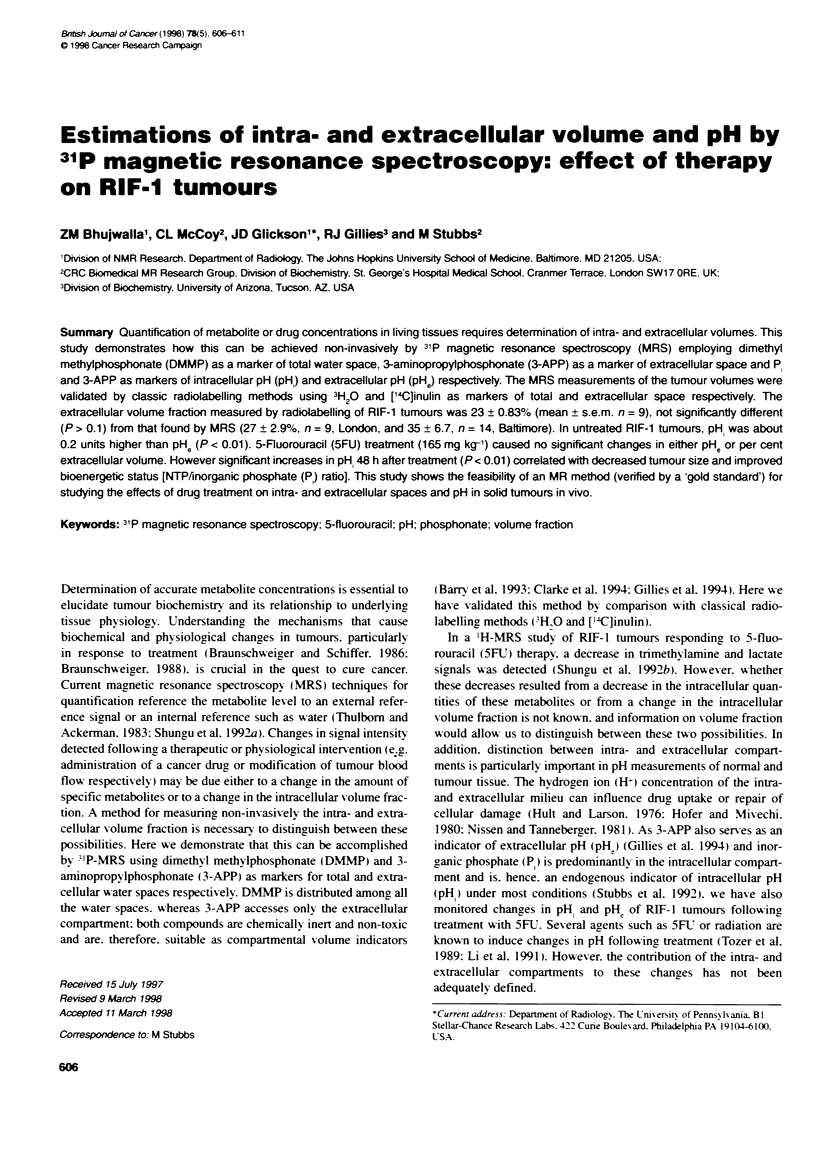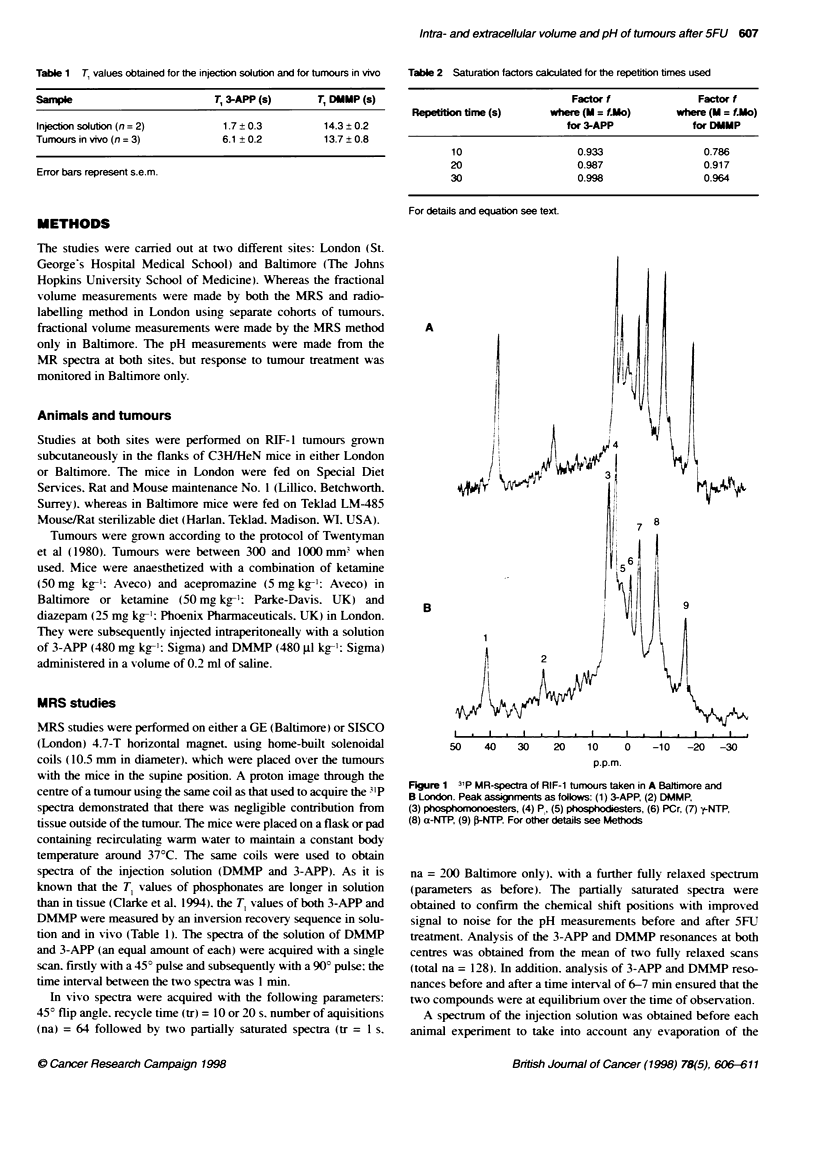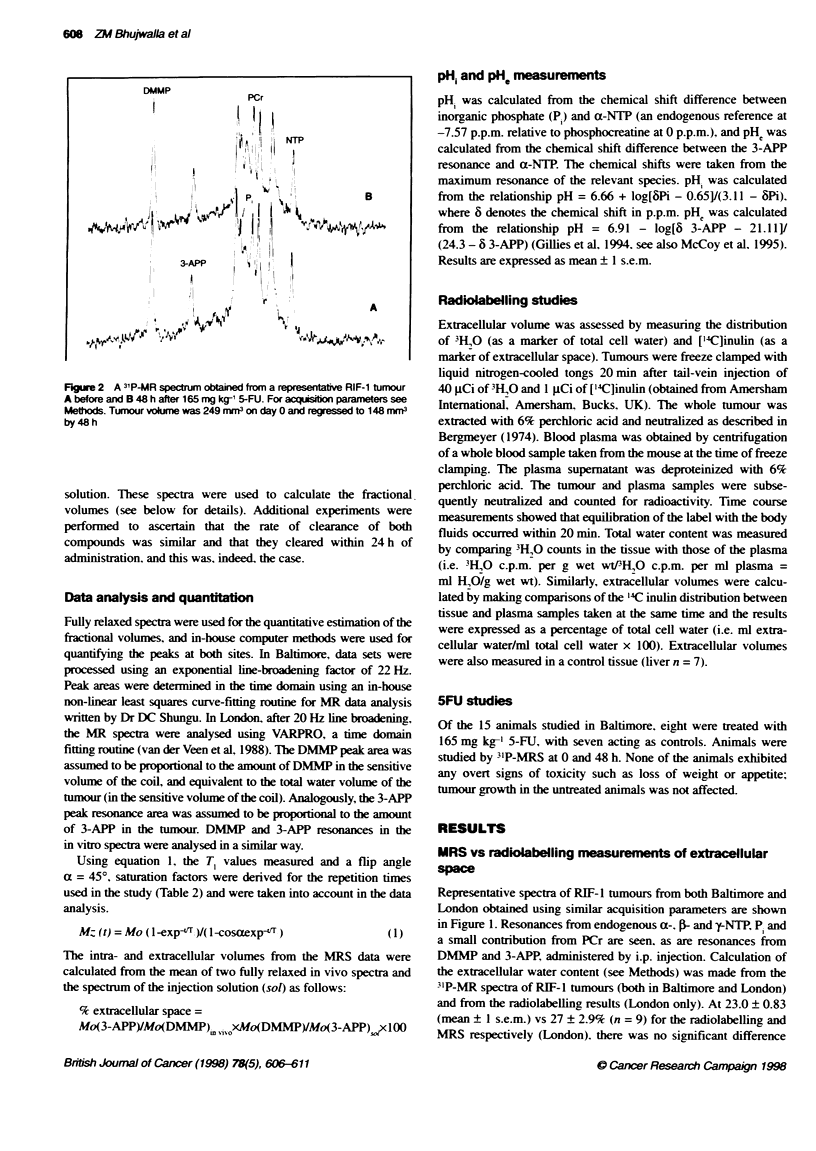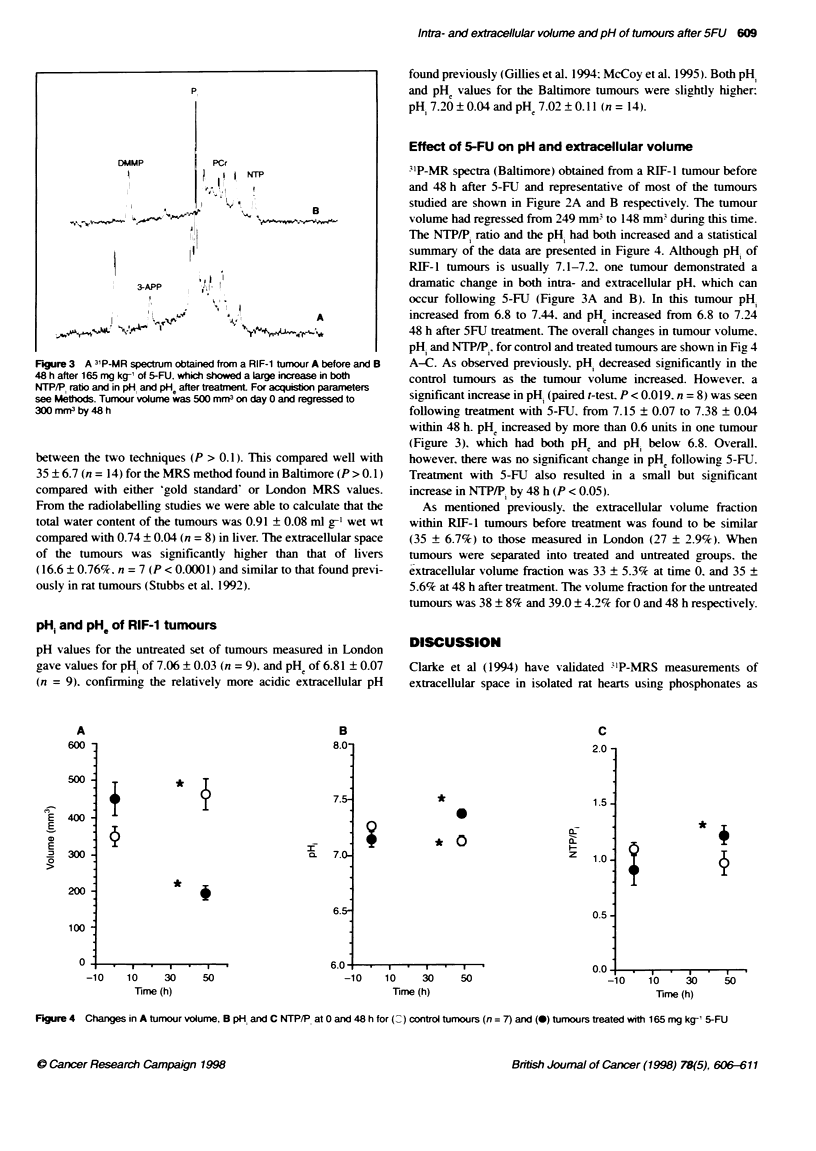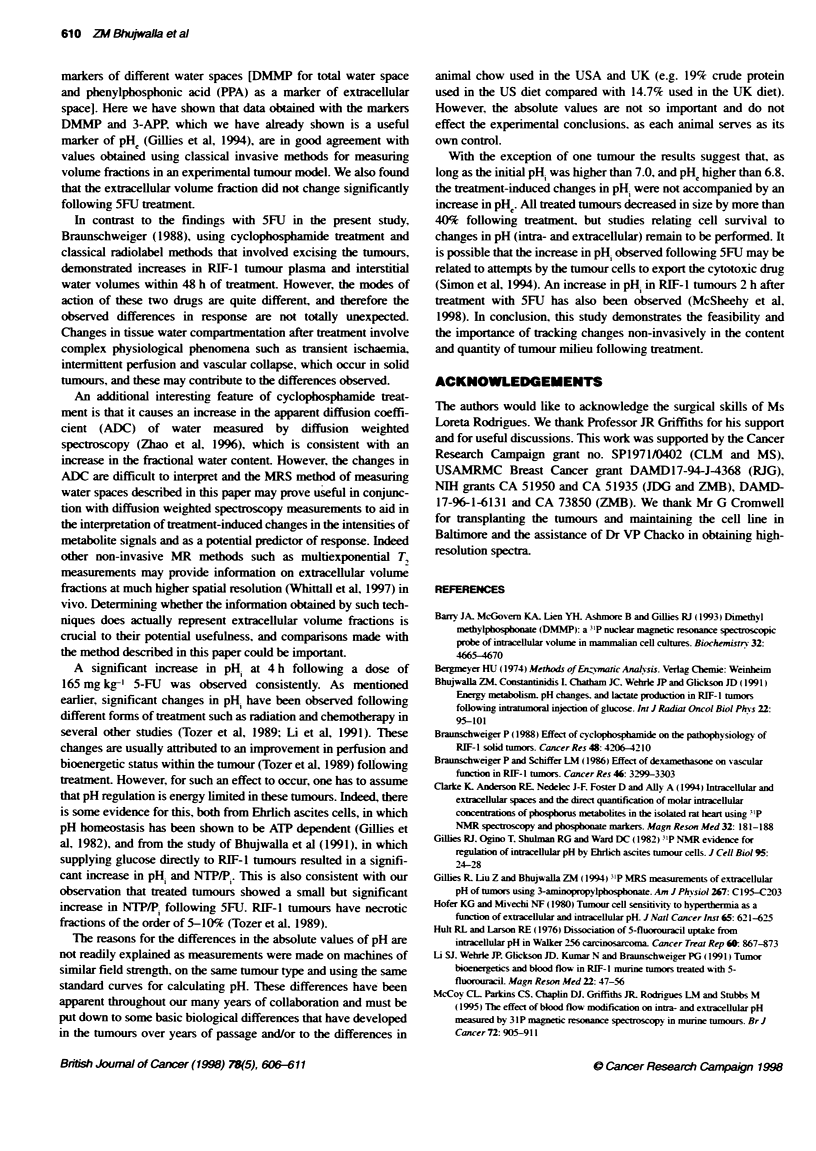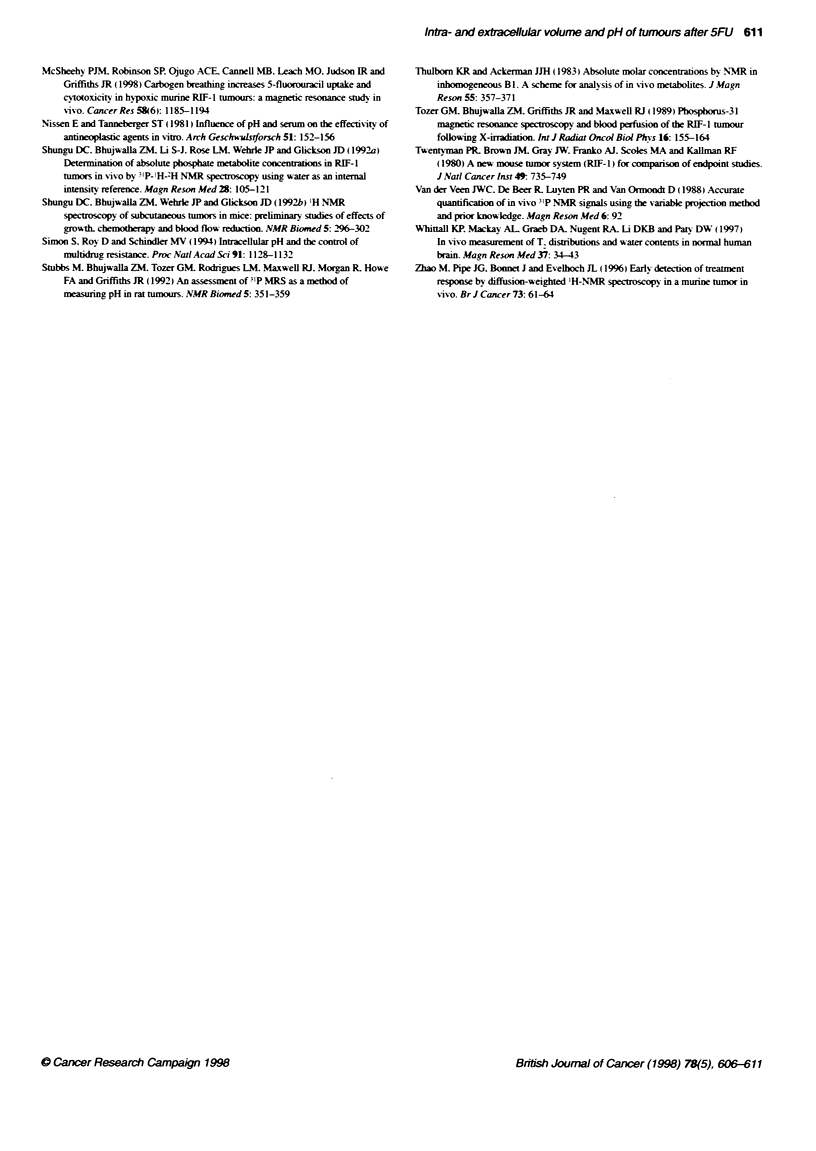# Estimations of intra- and extracellular volume and pH by 31P magnetic resonance spectroscopy: effect of therapy on RIF-1 tumours.

**DOI:** 10.1038/bjc.1998.548

**Published:** 1998-09

**Authors:** Z. M. Bhujwalla, C. L. McCoy, J. D. Glickson, R. J. Gillies, M. Stubbs

**Affiliations:** Department of Radiology, The Johns Hopkins University School of Medicine, Baltimore, MD 21205, USA.

## Abstract

Quantification of metabolite or drug concentrations in living tissues requires determination of intra- and extracellular volumes. This study demonstrates how this can be achieved non-invasively by 31P magnetic resonance spectroscopy (MRS) employing dimethyl methylphosphonate (DMMP) as a marker of total water space, 3-aminopropylphosphonate (3-APP) as a marker of extracellular space and P and 3-APP as markers of intracellular pH (pH) and extracellular pH (pHe) respectively. The MRS measurements of the tumour volumes were validated by classic radiolabelling methods using 3H2O and [14C]inulin as markers of total and extracellular space respectively. The extracellular volume fraction measured by radiolabelling of RIF-1 tumours was 23 +/- 0.83% (mean +/- s.e.m. n = 9), not significantly different (P > 0.1) from that found by MRS (27 +/- 2.9%, n = 9, London, and 35 +/- 6.7, n = 14, Baltimore). In untreated RIF-1 tumours, pH was about 0.2 units higher than pHe (P < 0.01). 5-Fluorouracil (5FU) treatment (165 mg kg(-1)) caused no significant changes in either pHe or per cent extracellular volume. However significant increases in pH, 48 h after treatment (P < 0.01) correlated with decreased tumour size and improved bioenergetic status [NTP/inorganic phosphate (Pi) ratio]. This study shows the feasibility of an MR method (verified by a 'gold standard') for studying the effects of drug treatment on intra- and extracellular spaces and pH in solid tumours in vivo.


British Journal of Cancer (1998) 78(5), 606–611
© 1998 Cancer Research Campaign

### Estimations of intra- and extracellular volume and pH by $^{31}P$ magnetic resonance spectroscopy: effect of therapy on RIF-1 tumours

**ZM Bhujwalla[1], CL McCoy[2], JD Glickson[1*], RJ Gillies[3] and M Stubbs[2]**

[1]Division of NMR Research, Department of Radiology, The Johns Hopkins University School of Medicine, Baltimore, MD 21205, USA;
[2]CRC Biomedical MR Research Group, Division of Biochemistry, St. George's Hospital Medical School, Cranmer Terrace, London SW17 0RE, UK;
[3]Division of Biochemistry, University of Arizona, Tucson, AZ, USA

**Summary** Quantification of metabolite or drug concentrations in living tissues requires determination of intra- and extracellular volumes. This study demonstrates how this can be achieved non-invasively by $^{31}P$ magnetic resonance spectroscopy (MRS) employing dimethyl methylphosphonate (DMMP) as a marker of total water space, 3-aminopropylphosphonate (3-APP) as a marker of extracellular space and $P_i$ and 3-APP as markers of intracellular pH ($pH_i$) and extracellular pH ($pH_e$) respectively. The MRS measurements of the tumour volumes were validated by classic radiolabelling methods using $^3H_2O$ and [$^{14}C$]inulin as markers of total and extracellular space respectively. The extracellular volume fraction measured by radiolabelling of RIF-1 tumours was 23 ± 0.83% (mean ± s.e.m. $n$ = 9), not significantly different ($P$ > 0.1) from that found by MRS (27 ± 2.9%, $n$ = 9, London, and 35 ± 6.7, $n$ = 14, Baltimore). In untreated RIF-1 tumours, $pH_i$ was about 0.2 units higher than $pH_e$ ($P$ < 0.01). 5-Fluorouracil (5FU) treatment (165 mg $kg^{-1}$) caused no significant changes in either $pH_e$ or per cent extracellular volume. However significant increases in $pH_i$ 48 h after treatment ($P$ < 0.01) correlated with decreased tumour size and improved bioenergetic status [NTP/inorganic phosphate ($P_i$) ratio]. This study shows the feasibility of an MR method (verified by a 'gold standard') for studying the effects of drug treatment on intra- and extracellular spaces and pH in solid tumours in vivo.

**Keywords:** $^{31}P$ magnetic resonance spectroscopy; 5-fluorouracil; pH; phosphonate; volume fraction

Determination of accurate metabolite concentrations is essential to elucidate tumour biochemistry and its relationship to underlying tissue physiology. Understanding the mechanisms that cause biochemical and physiological changes in tumours, particularly in response to treatment (Braunschweiger and Schiffer, 1986; Braunschweiger, 1988), is crucial in the quest to cure cancer. Current magnetic resonance spectroscopy (MRS) techniques for quantification reference the metabolite level to an external reference signal or an internal reference such as water (Thulborn and Ackerman, 1983; Shungu et al, 1992*a*). Changes in signal intensity detected following a therapeutic or physiological intervention (e.g. administration of a cancer drug or modification of tumour blood flow respectively) may be due either to a change in the amount of specific metabolites or to a change in the intracellular volume fraction. A method for measuring non-invasively the intra- and extracellular volume fraction is necessary to distinguish between these possibilities. Here we demonstrate that this can be accomplished by $^{31}P$-MRS using dimethyl methylphosphonate (DMMP) and 3-aminopropylphosphonate (3-APP) as markers for total and extracellular water spaces respectively. DMMP is distributed among all the water spaces, whereas 3-APP accesses only the extracellular compartment; both compounds are chemically inert and non-toxic and are, therefore, suitable as compartmental volume indicators (Barry et al, 1993; Clarke et al, 1994; Gillies et al, 1994). Here we have validated this method by comparison with classical radiolabelling methods ($^3H_2O$ and [$^{14}C$]inulin).

In a $^1H$-MRS study of RIF-1 tumours responding to 5-fluorouracil (5FU) therapy, a decrease in trimethylamine and lactate signals was detected (Shungu et al, 1992*b*). However, whether these decreases resulted from a decrease in the intracellular quantities of these metabolites or from a change in the intracellular volume fraction is not known, and information on volume fraction would allow us to distinguish between these two possibilities. In addition, distinction between intra- and extracellular compartments is particularly important in pH measurements of normal and tumour tissue. The hydrogen ion ($H^+$) concentration of the intra- and extracellular milieu can influence drug uptake or repair of cellular damage (Hult and Larson, 1976; Hofer and Mivechi, 1980; Nissen and Tanneberger, 1981). As 3-APP also serves as an indicator of extracellular pH ($pH_e$) (Gillies et al, 1994) and inorganic phosphate ($P_i$) is predominantly in the intracellular compartment and is, hence, an endogenous indicator of intracellular pH ($pH_i$) under most conditions (Stubbs et al, 1992), we have also monitored changes in $pH_i$ and $pH_e$ of RIF-1 tumours following treatment with 5FU. Several agents such as 5FU or radiation are known to induce changes in pH following treatment (Tozer et al, 1989; Li et al, 1991). However, the contribution of the intra- and extracellular compartments to these changes has not been adequately defined.

*Received 15 July 1997*
*Revised 9 March 1998*
*Accepted 11 March 1998*

*Correspondence to:* M Stubbs

**Current address:* Department of Radiology, The University of Pennsylvania, B1 Stellar-Chance Research Labs, 422 Curie Boulevard, Philadelphia PA 19104-6100, USA.

**Table 1** $T_1$ values obtained for the injection solution and for tumours in vivo

| Sample | $T_1$ 3-APP (s) | $T_1$ DMMP (s) |
|---|---|---|
| Injection solution ($n = 2$) | 1.7 ± 0.3 | 14.3 ± 0.2 |
| Tumours in vivo ($n = 3$) | 6.1 ± 0.2 | 13.7 ± 0.8 |

Error bars represent s.e.m.

**Table 2** Saturation factors calculated for the repetition times used

| Repetition time (s) | Factor *f* where (M = *f*.Mo) for 3-APP | Factor *f* where (M = *f*.Mo) for DMMP |
|---|---|---|
| 10 | 0.933 | 0.786 |
| 20 | 0.987 | 0.917 |
| 30 | 0.998 | 0.964 |

For details and equation see text.

### METHODS

The studies were carried out at two different sites: London (St. George's Hospital Medical School) and Baltimore (The Johns Hopkins University School of Medicine). Whereas the fractional volume measurements were made by both the MRS and radio-labelling method in London using separate cohorts of tumours, fractional volume measurements were made by the MRS method only in Baltimore. The pH measurements were made from the MR spectra at both sites, but response to tumour treatment was monitored in Baltimore only.

### Animals and tumours

Studies at both sites were performed on RIF-1 tumours grown subcutaneously in the flanks of C3H/HeN mice in either London or Baltimore. The mice in London were fed on Special Diet Services, Rat and Mouse maintenance No. 1 (Lillico, Betchworth, Surrey), whereas in Baltimore mice were fed on Teklad LM-485 Mouse/Rat sterilizable diet (Harlan, Teklad, Madison, WI, USA).

Tumours were grown according to the protocol of Twentyman et al (1980). Tumours were between 300 and 1000 $mm^3$ when used. Mice were anaesthetized with a combination of ketamine (50 mg $kg^{-1}$; Aveco) and acepromazine (5 mg $kg^{-1}$; Aveco) in Baltimore or ketamine (50 mg $kg^{-1}$; Parke-Davis, UK) and diazepam (25 mg $kg^{-1}$; Phoenix Pharmaceuticals, UK) in London. They were subsequently injected intraperitoneally with a solution of 3-APP (480 mg $kg^{-1}$; Sigma) and DMMP (480 μl $kg^{-1}$; Sigma) administered in a volume of 0.2 ml of saline.

### MRS studies

MRS studies were performed on either a GE (Baltimore) or SISCO (London) 4.7-T horizontal magnet, using home-built solenoidal coils (10.5 mm in diameter), which were placed over the tumours with the mice in the supine position. A proton image through the centre of a tumour using the same coil as that used to acquire the $^{31}P$ spectra demonstrated that there was negligible contribution from tissue outside of the tumour. The mice were placed on a flask or pad containing recirculating warm water to maintain a constant body temperature around 37°C. The same coils were used to obtain spectra of the injection solution (DMMP and 3-APP). As it is known that the $T_1$ values of phosphonates are longer in solution than in tissue (Clarke et al, 1994), the $T_1$ values of both 3-APP and DMMP were measured by an inversion recovery sequence in solution and in vivo (Table 1). The spectra of the solution of DMMP and 3-APP (an equal amount of each) were acquired with a single scan, firstly with a 45° pulse and subsequently with a 90° pulse; the time interval between the two spectra was 1 min.

In vivo spectra were acquired with the following parameters: 45° flip angle, recycle time (tr) = 10 or 20 s, number of aquisitions (na) = 64 followed by two partially saturated spectra (tr = 1 s, na = 200 Baltimore only), with a further fully relaxed spectrum (parameters as before). The partially saturated spectra were obtained to confirm the chemical shift positions with improved signal to noise for the pH measurements before and after 5FU treatment. Analysis of the 3-APP and DMMP resonances at both centres was obtained from the mean of two fully relaxed scans (total na = 128). In addition, analysis of 3-APP and DMMP resonances before and after a time interval of 6–7 min ensured that the two compounds were at equilibrium over the time of observation.

A spectrum of the injection solution was obtained before each animal experiment to take into account any evaporation of the

**Figure 1** $^{31}P$ MR-spectra of RIF-1 tumours taken in **A** Baltimore and **B** London. Peak assignments as follows: (1) 3-APP, (2) DMMP, (3) phosphomonoesters, (4) $P_i$, (5) phosphodiesters, (6) PCr, (7) γ-NTP, (8) α-NTP, (9) β-NTP. For other details see Methods

**Figure 2** A $^{31}$P-MR spectrum obtained from a representative RIF-1 tumour **A** before and **B** 48 h after 165 mg $kg^{-1}$ 5-FU. For acquisition parameters see Methods. Tumour volume was 249 $mm^3$ on day 0 and regressed to 148 $mm^3$ by 48 h solution. These spectra were used to calculate the fractional volumes (see below for details). Additional experiments were performed to ascertain that the rate of clearance of both compounds was similar and that they cleared within 24 h of administration, and this was, indeed, the case.

### Data analysis and quantitation

Fully relaxed spectra were used for the quantitative estimation of the fractional volumes, and in-house computer methods were used for quantifying the peaks at both sites. In Baltimore, data sets were processed using an exponential line-broadening factor of 22 Hz. Peak areas were determined in the time domain using an in-house non-linear least squares curve-fitting routine for MR data analysis written by Dr DC Shungu. In London, after 20 Hz line broadening, the MR spectra were analysed using VARPRO, a time domain fitting routine (van der Veen et al, 1988). The DMMP peak area was assumed to be proportional to the amount of DMMP in the sensitive volume of the coil, and equivalent to the total water volume of the tumour (in the sensitive volume of the coil). Analogously, the 3-APP peak resonance area was assumed to be proportional to the amount of 3-APP in the tumour. DMMP and 3-APP resonances in the in vitro spectra were analysed in a similar way.

Using equation 1, the $T_1$ values measured and a flip angle $\alpha = 45°$, saturation factors were derived for the repetition times used in the study (Table 2) and were taken into account in the data analysis.

$$Mz\,(t) = Mo\,(1\text{-exp}^{-t/T})/(1\text{-cos}\alpha\,\text{exp}^{-t/T}) \qquad (1)$$

The intra- and extracellular volumes from the MRS data were calculated from the mean of two fully relaxed in vivo spectra and the spectrum of the injection solution (*sol*) as follows:

% extracellular space =

$$Mo(\text{3-APP})/Mo(\text{DMMP})_{\text{in vivo}} \times Mo(\text{DMMP})/Mo(\text{3-APP})_{sol} \times 100$$

### $pH_i$ and $pH_e$ measurements

$pH_i$ was calculated from the chemical shift difference between inorganic phosphate ($P_i$) and α-NTP (an endogenous reference at –7.57 p.p.m. relative to phosphocreatine at 0 p.p.m.), and $pH_e$ was calculated from the chemical shift difference between the 3-APP resonance and α-NTP. The chemical shifts were taken from the maximum resonance of the relevant species. $pH_i$ was calculated from the relationship pH = 6.66 + log[δPi – 0.65]/(3.11 – δPi), where δ denotes the chemical shift in p.p.m. $pH_e$ was calculated from the relationship pH = 6.91 – log[δ 3-APP – 21.11]/ (24.3 – δ 3-APP) (Gillies et al, 1994, see also McCoy et al, 1995). Results are expressed as mean ± 1 s.e.m.

### Radiolabelling studies

Extracellular volume was assessed by measuring the distribution of $^3H_2O$ (as a marker of total cell water) and [$^{14}$C]inulin (as a marker of extracellular space). Tumours were freeze clamped with liquid nitrogen-cooled tongs 20 min after tail-vein injection of 40 μCi of $^3H_2O$ and 1 μCi of [$^{14}$C]inulin (obtained from Amersham International, Amersham, Bucks, UK). The whole tumour was extracted with 6% perchloric acid and neutralized as described in Bergmeyer (1974). Blood plasma was obtained by centrifugation of a whole blood sample taken from the mouse at the time of freeze clamping. The plasma supernatant was deproteinized with 6% perchloric acid. The tumour and plasma samples were subsequently neutralized and counted for radioactivity. Time course measurements showed that equilibration of the label with the body fluids occurred within 20 min. Total water content was measured by comparing $^3H_2O$ counts in the tissue with those of the plasma (i.e. $^3H_2O$ c.p.m. per g wet wt/$^3H_2O$ c.p.m. per ml plasma = ml $H_2O$/g wet wt). Similarly, extracellular volumes were calculated by making comparisons of the $^{14}$C inulin distribution between tissue and plasma samples taken at the same time and the results were expressed as a percentage of total cell water (i.e. ml extracellular water/ml total cell water × 100). Extracellular volumes were also measured in a control tissue (liver $n = 7$).

### 5FU studies

Of the 15 animals studied in Baltimore, eight were treated with 165 mg $kg^{-1}$ 5-FU, with seven acting as controls. Animals were studied by $^{31}$P-MRS at 0 and 48 h. None of the animals exhibited any overt signs of toxicity such as loss of weight or appetite; tumour growth in the untreated animals was not affected.

### RESULTS

### MRS vs radiolabelling measurements of extracellular space

Representative spectra of RIF-1 tumours from both Baltimore and London obtained using similar acquisition parameters are shown in Figure 1. Resonances from endogenous α-, β- and γ-NTP, $P_i$ and a small contribution from PCr are seen, as are resonances from DMMP and 3-APP, administered by i.p. injection. Calculation of the extracellular water content (see Methods) was made from the $^{31}$P-MR spectra of RIF-1 tumours (both in Baltimore and London) and from the radiolabelling results (London only). At 23.0 ± 0.83 (mean ± 1 s.e.m.) vs 27 ± 2.9% ($n = 9$) for the radiolabelling and MRS respectively (London), there was no significant difference

© Cancer Research Campaign 1998

**Figure 3** A $^{31}$P-MR spectrum obtained from a RIF-1 tumour **A** before and **B** 48 h after 165 mg kg$^{-1}$ of 5-FU, which showed a large increase in both NTP/$P_i$ ratio and in $pH_i$ and $pH_e$ after treatment. For acquistion parameters see Methods. Tumour volume was 500 mm$^3$ on day 0 and regressed to 300 mm$^3$ by 48 h between the two techniques ($P > 0.1$). This compared well with $35 \pm 6.7$ ($n = 14$) for the MRS method found in Baltimore ($P > 0.1$) compared with either 'gold standard' or London MRS values. From the radiolabelling studies we were able to calculate that the total water content of the tumours was $0.91 \pm 0.08$ ml g$^{-1}$ wet wt compared with $0.74 \pm 0.04$ ($n = 8$) in liver. The extracellular space of the tumours was significantly higher than that of livers ($16.6 \pm 0.76\%$, $n = 7$ ($P < 0.0001$) and similar to that found previously in rat tumours (Stubbs et al, 1992).

### $pH_i$ and $pH_e$ of RIF-1 tumours pH values for the untreated set of tumours measured in London gave values for $pH_i$ of $7.06 \pm 0.03$ ($n = 9$), and $pH_e$ of $6.81 \pm 0.07$ ($n = 9$), confirming the relatively more acidic extracellular pH found previously (Gillies et al, 1994; McCoy et al, 1995). Both $pH_i$ and $pH_e$ values for the Baltimore tumours were slightly higher: $pH_i$ $7.20 \pm 0.04$ and $pH_e$ $7.02 \pm 0.11$ ($n = 14$).

### Effect of 5-FU on pH and extracellular volume

$^{31}$P-MR spectra (Baltimore) obtained from a RIF-1 tumour before and 48 h after 5-FU and representative of most of the tumours studied are shown in Figure 2A and B respectively. The tumour volume had regressed from 249 mm$^3$ to 148 mm$^3$ during this time. The NTP/$P_i$ ratio and the $pH_i$ had both increased and a statistical summary of the data are presented in Figure 4. Although $pH_i$ of RIF-1 tumours is usually 7.1–7.2, one tumour demonstrated a dramatic change in both intra- and extracellular pH, which can occur following 5-FU (Figure 3A and B). In this tumour $pH_i$ increased from 6.8 to 7.44, and $pH_e$ increased from 6.8 to 7.24 48 h after 5FU treatment. The overall changes in tumour volume, $pH_i$ and NTP/$P_i$, for control and treated tumours are shown in Fig 4 A–C. As observed previously, $pH_i$ decreased significantly in the control tumours as the tumour volume increased. However, a significant increase in $pH_i$ (paired *t*-test, $P < 0.019$, $n = 8$) was seen following treatment with 5-FU, from $7.15 \pm 0.07$ to $7.38 \pm 0.04$ within 48 h. $pH_e$ increased by more than 0.6 units in one tumour (Figure 3), which had both $pH_e$ and $pH_i$ below 6.8. Overall, however, there was no significant change in $pH_e$ following 5-FU. Treatment with 5-FU also resulted in a small but significant increase in NTP/$P_i$ by 48 h ($P < 0.05$).

As mentioned previously, the extracellular volume fraction within RIF-1 tumours before treatment was found to be similar ($35 \pm 6.7\%$) to those measured in London ($27 \pm 2.9\%$). When tumours were separated into treated and untreated groups, the extracellular volume fraction was $33 \pm 5.3\%$ at time 0, and $35 \pm 5.6\%$ at 48 h after treatment. The volume fraction for the untreated tumours was $38 \pm 8\%$ and $39.0 \pm 4.2\%$ for 0 and 48 h respectively.

### DISCUSSION

Clarke et al (1994) have validated $^{31}$P-MRS measurements of extracellular space in isolated rat hearts using phosphonates as

**Figure 4** Changes in **A** tumour volume, **B** $pH_i$ and **C** NTP/$P_i$ at 0 and 48 h for (○) control tumours ($n = 7$) and (●) tumours treated with 165 mg kg$^{-1}$ 5-FU

markers of different water spaces [DMMP for total water space and phenylphosphonic acid (PPA) as a marker of extracellular space]. Here we have shown that data obtained with the markers DMMP and 3-APP, which we have already shown is a useful marker of $pH_e$ (Gillies et al, 1994), are in good agreement with values obtained using classical invasive methods for measuring volume fractions in an experimental tumour model. We also found that the extracellular volume fraction did not change significantly following 5FU treatment.

In contrast to the findings with 5FU in the present study, Braunschweiger (1988), using cyclophosphamide treatment and classical radiolabel methods that involved excising the tumours, demonstrated increases in RIF-1 tumour plasma and interstitial water volumes within 48 h of treatment. However, the modes of action of these two drugs are quite different, and therefore the observed differences in response are not totally unexpected. Changes in tissue water compartmentation after treatment involve complex physiological phenomena such as transient ischaemia, intermittent perfusion and vascular collapse, which occur in solid tumours, and these may contribute to the differences observed.

An additional interesting feature of cyclophosphamide treatment is that it causes an increase in the apparent diffusion coefficient (ADC) of water measured by diffusion weighted spectroscopy (Zhao et al, 1996), which is consistent with an increase in the fractional water content. However, the changes in ADC are difficult to interpret and the MRS method of measuring water spaces described in this paper may prove useful in conjunction with diffusion weighted spectroscopy measurements to aid in the interpretation of treatment-induced changes in the intensities of metabolite signals and as a potential predictor of response. Indeed other non-invasive MR methods such as multiexponential $T_2$ measurements may provide information on extracellular volume fractions at much higher spatial resolution (Whittall et al, 1997) in vivo. Determining whether the information obtained by such techniques does actually represent extracellular volume fractions is crucial to their potential usefulness, and comparisons made with the method described in this paper could be important.

A significant increase in $pH_i$ at 4 h following a dose of 165 mg $kg^{-1}$ 5-FU was observed consistently. As mentioned earlier, significant changes in $pH_i$ have been observed following different forms of treatment such as radiation and chemotherapy in several other studies (Tozer et al, 1989; Li et al, 1991). These changes are usually attributed to an improvement in perfusion and bioenergetic status within the tumour (Tozer et al, 1989) following treatment. However, for such an effect to occur, one has to assume that pH regulation is energy limited in these tumours. Indeed, there is some evidence for this, both from Ehrlich ascites cells, in which pH homeostasis has been shown to be ATP dependent (Gillies et al, 1982), and from the study of Bhujwalla et al (1991), in which supplying glucose directly to RIF-1 tumours resulted in a significant increase in $pH_i$ and $NTP/P_i$. This is also consistent with our observation that treated tumours showed a small but significant increase in $NTP/P_i$ following 5FU. RIF-1 tumours have necrotic fractions of the order of 5–10% (Tozer et al, 1989).

The reasons for the differences in the absolute values of pH are not readily explained as measurements were made on machines of similar field strength, on the same tumour type and using the same standard curves for calculating pH. These differences have been apparent throughout our many years of collaboration and must be put down to some basic biological differences that have developed in the tumours over years of passage and/or to the differences in animal chow used in the USA and UK (e.g. 19% crude protein used in the US diet compared with 14.7% used in the UK diet). However, the absolute values are not so important and do not effect the experimental conclusions, as each animal serves as its own control.

With the exception of one tumour the results suggest that, as long as the initial $pH_i$ was higher than 7.0, and $pH_e$ higher than 6.8, the treatment-induced changes in $pH_i$ were not accompanied by an increase in $pH_e$. All treated tumours decreased in size by more than 40% following treatment, but studies relating cell survival to changes in pH (intra- and extracellular) remain to be performed. It is possible that the increase in $pH_i$ observed following 5FU may be related to attempts by the tumour cells to export the cytotoxic drug (Simon et al, 1994). An increase in $pH_i$ in RIF-1 tumours 2 h after treatment with 5FU has also been observed (McSheehy et al, 1998). In conclusion, this study demonstrates the feasibility and the importance of tracking changes non-invasively in the content and quantity of tumour milieu following treatment.

### ACKNOWLEDGEMENTS

The authors would like to acknowledge the surgical skills of Ms Loreta Rodrigues. We thank Professor JR Griffiths for his support and for useful discussions. This work was supported by the Cancer Research Campaign grant no. SP1971/0402 (CLM and MS), USAMRMC Breast Cancer grant DAMD17-94-J-4368 (RJG), NIH grants CA 51950 and CA 51935 (JDG and ZMB), DAMD-17-96-1-6131 and CA 73850 (ZMB). We thank Mr G Cromwell for transplanting the tumours and maintaining the cell line in Baltimore and the assistance of Dr VP Chacko in obtaining high-resolution spectra.

### REFERENCES

Barry JA, McGovern KA, Lien YH, Ashmore B and Gillies RJ (1993) Dimethyl methylphosphonate (DMMP): a [31]P nuclear magnetic resonance spectroscopic probe of intracellular volume in mammalian cell cultures. *Biochemistry* **32**: 4665–4670

Bergmeyer HU (1974) *Methods of Enzymatic Analysis*. Verlag Chemie: Weinheim

Bhujwalla ZM, Constantinidis I, Chatham JC, Wehrle JP and Glickson JD (1991) Energy metabolism, pH changes, and lactate production in RIF-1 tumors following intratumoral injection of glucose. *Int J Radiat Oncol Biol Phys* **22**: 95–101

Braunschweiger P (1988) Effect of cyclophosphamide on the pathophysiology of RIF-1 solid tumors. *Cancer Res* **48**: 4206–4210

Braunschweiger P and Schiffer LM (1986) Effect of dexamethasone on vascular function in RIF-1 tumors. *Cancer Res* **46**: 3299–3303

Clarke K, Anderson RE, Nedelec J-F, Foster D and Ally A (1994) Intracellular and extracellular spaces and the direct quantification of molar intracellular concentrations of phosphorus metabolites in the isolated rat heart using [31]P NMR spectroscopy and phosphonate markers. *Magn Reson Med* **32**: 181–188

Gillies RJ, Ogino T, Shulman RG and Ward DC (1982) [31]P NMR evidence for regulation of intracellular pH by Ehrlich ascites tumour cells. *J Cell Biol* **95**: 24–28

Gillies R, Liu Z and Bhujwalla ZM (1994) [31]P MRS measurements of extracellular pH of tumors using 3-aminopropylphosphonate. *Am J Physiol* **267**: C195–C203

Hofer KG and Mivechi NF (1980) Tumour cell sensitivity to hyperthermia as a function of extracellular and intracellular pH. *J Natl Cancer Inst* **65**: 621–625

Hult RL and Larson RE (1976) Dissociation of 5-fluorouracil uptake from intracellular pH in Walker 256 carcinosarcoma. *Cancer Treat Rep* **60**: 867–873

Li SJ, Wehrle JP, Glickson JD, Kumar N and Braunschweiger PG (1991) Tumor bioenergetics and blood flow in RIF-1 murine tumors treated with 5-fluorouracil. *Magn Reson Med* **22**: 47–56

McCoy CL, Parkins CS, Chaplin DJ, Griffiths JR, Rodrigues LM and Stubbs M (1995) The effect of blood flow modification on intra- and extracellular pH measured by 31P magnetic resonance spectroscopy in murine tumours. *Br J Cancer* **72**: 905–911

© Cancer Research Campaign 1998

McSheehy PJM, Robinson SP, Ojugo ACE, Cannell MB, Leach MO, Judson IR and Griffiths JR (1998) Carbogen breathing increases 5-fluorouracil uptake and cytotoxicity in hypoxic murine RIF-1 tumours: a magnetic resonance study in vivo. *Cancer Res* **58**(6): 1185–1194

Nissen E and Tanneberger ST (1981) Influence of pH and serum on the effectivity of antineoplastic agents in vitro. *Arch Geschwulstforsch* **51**: 152–156

Shungu DC, Bhujwalla ZM, Li S-J, Rose LM, Wehrle JP and Glickson JD (1992*a*) Determination of absolute phosphate metabolite concentrations in RIF-1 tumors in vivo by $^{31}$P-$^{1}$H-$^{2}$H NMR spectroscopy using water as an internal intensity reference. *Magn Reson Med* **28**: 105–121

Shungu DC, Bhujwalla ZM, Wehrle JP and Glickson JD (1992*b*) $^{1}$H NMR spectroscopy of subcutaneous tumors in mice: preliminary studies of effects of growth, chemotherapy and blood flow reduction. *NMR Biomed* **5**: 296–302

Simon S, Roy D and Schindler MV (1994) Intracellular pH and the control of multidrug resistance. *Proc Natl Acad Sci* **91**: 1128–1132

Stubbs M, Bhujwalla ZM, Tozer GM, Rodrigues LM, Maxwell RJ, Morgan R, Howe FA and Griffiths JR (1992) An assessment of $^{31}$P MRS as a method of measuring pH in rat tumours. *NMR Biomed* **5**: 351–359

Thulborn KR and Ackerman JJH (1983) Absolute molar concentrations by NMR in inhomogeneous B1. A scheme for analysis of in vivo metabolites. *J Magn Reson* **55**: 357–371

Tozer GM, Bhujwalla ZM, Griffiths JR and Maxwell RJ (1989) Phosphorus-31 magnetic resonance spectroscopy and blood perfusion of the RIF-1 tumour following X-irradiation. *Int J Radiat Oncol Biol Phys* **16**: 155–164

Twentyman PR, Brown JM, Gray JW, Franko AJ, Scoles MA and Kallman RF (1980) A new mouse tumor system (RIF-1) for comparison of endpoint studies. *J Natl Cancer Inst* **49**: 735–749

Van der Veen JWC, De Beer R, Luyten PR and Van Ormondt D (1988) Accurate quantification of in vivo $^{31}$P NMR signals using the variable projection method and prior knowledge. *Magn Reson Med* **6**: 92

Whittall KP, Mackay AL, Graeb DA, Nugent RA, Li DKB and Paty DW (1997) In vivo measurement of $T_2$ distributions and water contents in normal human brain. *Magn Reson Med* **37**: 34–43

Zhao M, Pipe JG, Bonnet J and Evelhoch JL (1996) Early detection of treatment response by diffusion-weighted $^{1}$H-NMR spectroscopy in a murine tumor in vivo. *Br J Cancer* **73**: 61–64

© Cancer Research Campaign 1998